# Editorial: Enterobacteriaceae Antimicrobial Agents and Resistance: Relationship With the Therapeutic Approach

**DOI:** 10.3389/fcimb.2021.728371

**Published:** 2021-09-14

**Authors:** Maria Teresa Mascellino, Silpak Biswas, Alessandra Oliva

**Affiliations:** ^1^Department of Public Health and Infectious Diseases, Sapienza University, Rome, Italy; ^2^Department of Microbiology, School of Tropical Medicine, Kolkata, India

**Keywords:** multi-drug resistant *Enterobacterales*, innovative therapies, association of antibiotics, plasmids, biofilm, phages

In the field of infectious diseases multidrug-resistant (MDR) Gram-negative bacteria such as *Enterobacterales*, *Acinetobacter baumannii*, *Pseudomonas aeruginosa*, and *Aeromonas hydrophila* constitute an important issue for establishing correct and appropriate therapies in patients admitted to both Intensive Care Units and General Medicine centers including respiratory care wards. Carbapenem-resistant *Enterobacterales* (CRE) have undergone extensive dissemination worldwide, resulting in increased mortality and a global threat to public health. Additionally, the predominant production of *Klebsiella pneumoniae* carbapenemase (KPC) contributed to the most important mechanism of carbapenem resistance in *K. pneumoniae* (Nordmann et al., 2009).

To deal with this situation it is necessary to understand the infection epidemiology and the resistance patterns other than those that comply with guidelines for treatment (Kaye and Pogue, 2015). The development of new drugs capable of eradicating multidrug-resistant Gram-negative pathogens is highly recommended as is combination therapy (which is more effective than monotherapy) or the research into other alternatives (**Figure 1**) (Morris and Cerceo, 2020).

**Figure 1:**
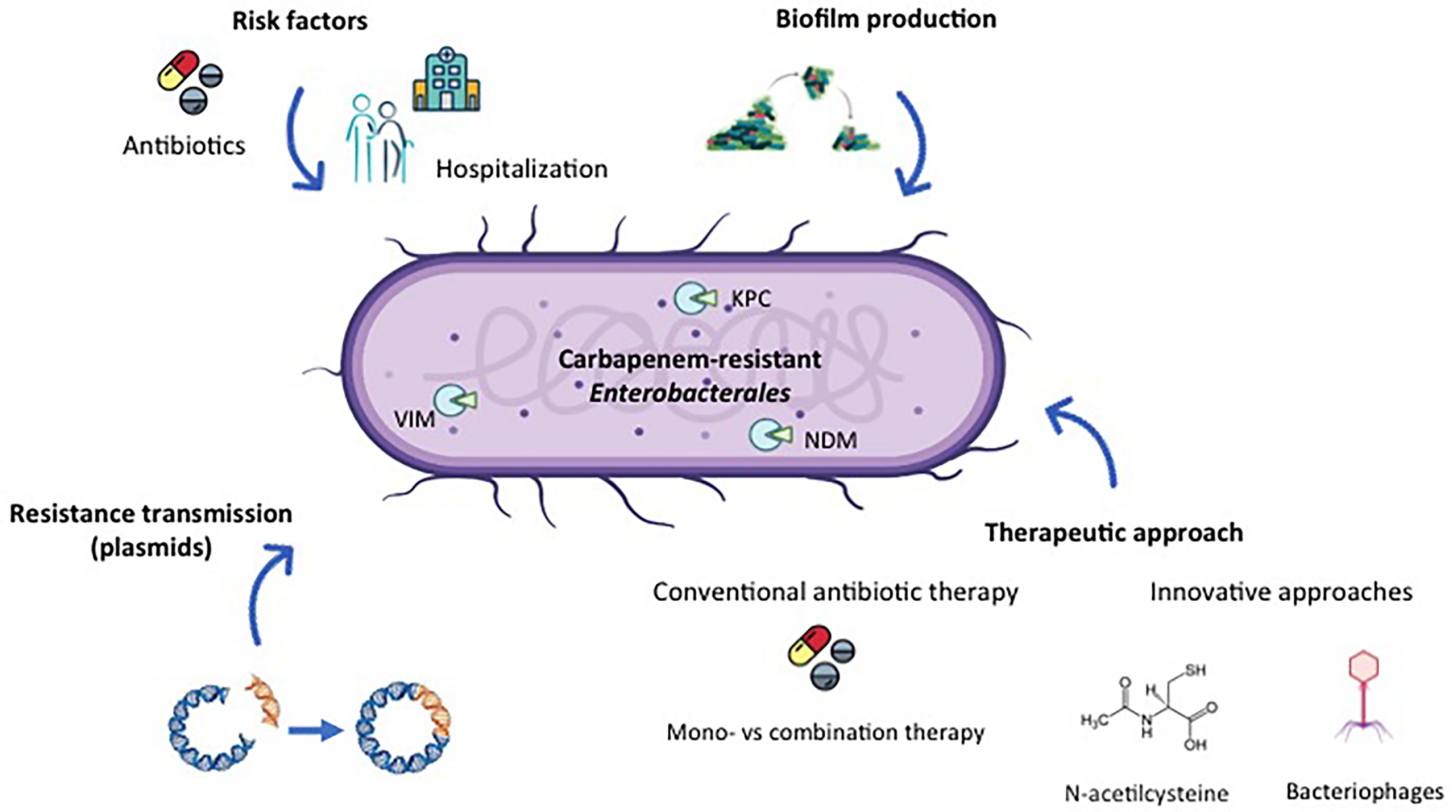
Overview of different features of Carbapenem-Resistant *Enterobacterales (CRE)* management.

The topics covered by this Research Topic include contributions on the dissemination and characteristics of carbapenemases such as KPC, NDM, OXA 48, IMP, and VIM among CRE, which are crucial for detecting resistance in adult or child patients. These concepts are well analyzed by the Antimicrobial Surveillance Network (CHINET) Study Group in China (Han et al.).

In this large study, carbapenemases were found in 97.4% of CRE strains, including KPC-2 (51.6%), NDM (35.7%), and OXA-48-like carbapenemases (7.3%). The most prevalent carbapenemase genes were *blaKPC-2* among *K. pneumoniae* isolates from adult patients, and *blaNDM* among *E. coli* isolates from children. All the CRE strains were highly resistant to cephalosporins and carbapenems. The risk factors and the epidemiology for the establishment of carbapenem-resistant *Klebsiella pneumoniae* (CRKP) are issues widely reported in literature on this subject (Pitout et al., 2015; Logan and Weinstein, 2017; Pin et al., 2018).

These concepts are highlighted in other studies performed in China (Fang et al.). Here, the colonization and the incidence of infections were reported to be 2.7 per 100,000 patient days, and the presence of CRKP KPC-2 was found predominantly. The MALDI-TOF of MS system was shown to be useful for detecting this prevailing serotype with the same performance as the PFGE (Pulsed-Field Gel Electrophoresis) system. The mainstream gene of CRKP in the geographic areas of China was *blaKPC−*2.

Another paper also found that there was a predominance of KPC-2. In this case, the mobile elements such as plasmids were detected and accurately studied. CRKP strains co-harboring *blaKPC-2*-carrying plasmid and pLVPK-like virulence plasmid are mostly involved in bacterial multidrug resistance, enhanced virulence, and above all, in the transfer of these mobile elements to other bacteria such as *E. coli* and other isolates (Du et al.).

A very interesting article concentrates on the presence of plasmids and concerns a new unusual non–carbapenemase-producing CRKP carrying a rare plasmid-borne inducible AmpC gene, *blaDHA-1* from an isolate belonging to blood culture. This strain showed complex susceptibility patterns. The genetic method Whole Genome Sequencing was able to detect this peculiar plasmid bearing the resistance genes of the third generation cephalosporins (Realegeno et al.).

The early detection of the third generation cephalosporins resistant *Enterobacterales* directly from positive blood cultures is very important in identifying antimicrobial resistance before the culture, strongly shortening the time for the establishment of a correct and appropriate antibiotic therapy (Durand et al.). For this purpose, two methods (electrochemical and chromogenic) were proposed by Hospital in Nimes (France) and are discussed in comparison with the traditional technology, which consequently allows for a rapid adaptation of therapy, with great benefits for patient outcome.

The production of bacterial biofilm is another big inconvenience (**Figure 1**). The microorganisms universally attach to surfaces and produce extracellular polysaccharides, resulting in biofilm formation (Donlan, 2001). This process especially occurs on medical devices (Oliva et al., 2013). The presence of biofilm together with other factors related to virulence is a significant risk factor, especially in oncological patients, as demonstrated in a study performed in a hospital in Rome (Italy). The detection of the biofilm in these high-risk patients may be of help in the management of oncological individuals (Di Domenico et al.).

Other than *Enterobacterales*, other microorganisms such as *Aeromonas hydrophila*, which lives in an aquatic environment and rarely infects individuals, are found to own chromosomally encoded carbapenem resistant genes, such as *blacphA7* (metallo-beta-lactamase). Consequently, the emerging MDR *Aeromonas* should also be taken into account. The first case of CphA-mediated carbapenem resistant *A. hydrophila* was reported in the U.S. (Hilt et al.).

Different ST (Sequence Types) have been found among resistant *Enterobacterales*, especially CRKP. Even though the mortality rate was detected and shown to be no different because of the diverse ST, other factors affected mortality, such as the treatment strategies following source control and bacterial eradication (Lim et al.). Furthermore, the mortality resulted as being increased in long-term facilities such as residences for the elderly or hospices, which achieved a rate of up to 75% (Chen et al.).

Besides phagotherapy (Principi et al., 2019) and possible antimicrobial combinations, including non-antibiotic compounds such as N-acetylcysteine (MacNair et al., 2018: Oliva et al., 2021), there are two crucial tools for overcoming microbial multidrug resistance and consequently decreasing the great menace of this issue to public health (**Figure 1**). Bacteriophages are expected to become a potentially effective therapeutic agent for difficult-to-treat infections. In this case, a specific bacteriophage against a particular microorganism could be used to lyse and kill the bacterium infected by the phage. Unlike antibiotics, in this situation, no resistance occurs. The limit of phage therapy, inherent to bacteriophage, lies in a narrow spectrum of action. In fact, in the case reported in this Special Issue concerning a mixed infection, a failure of three consecutive phage therapies was reported. However, a set of different bacteriophages were selected against the single bacterial strains involved in the infection, leading to a successful patient outcome (Qin et al.).

Interestingly, combination therapy seems to be more beneficial than monotherapy as far as mortality rates are concerned. Many antibiotics are used in association against CRE, such as colistin-based regimens whereas data on ceftazidime/avibactam used in combination or alone are still conflicting.

Even the association of old antibiotics such as nitrofurantoin and amikacin appears to be effective against 12 clinical MDR uropathogenic *E. coli in vitro*, as shown by an article in this Research Topic (Zhong et al.).

In conclusion, the multi-resistance in *Enterobacterales* and other Gram-negative bacteria is an increasing problem that requires a drastic intervention for limiting the further spread of resistant bacteria. Alternative therapeutic programs other than antibiotics could be proposed, such as medicinal oils, antibodies, common biocides, killing factors, and phage therapy.

## Author Contributions

MTM conceived, organized and wrote the Editorial. SB contributed to the revision of articles concerning the epidemiology and the risk factors. AO contributed to the revision of articles concerning the production of the biofilm and the antibiotics association. All authors contributed to the article and approved the submitted version.

## Conflict of Interest

The authors declare that the research was conducted in the absence of any commercial or financial relationships that could be construed as a potential conflict of interest.

## Publisher’s Note

All claims expressed in this article are solely those of the authors and do not necessarily represent those of their affiliated organizations, or those of the publisher, the editors and the reviewers. Any product that may be evaluated in this article, or claim that may be made by its manufacturer, is not guaranteed or endorsed by the publisher.

## Abbreviations

KPC, *Klebsiella pneumoniae* carbapenemase producing; NDM, New Delhi metallo-beta-lactamase; VIM, Verona Integron-encoded Metallo-β-lactamase.

## References

[B1] Donlan L. M. (2001). Biofilm Formation: A Clinically Relevant Microbiological Process. Clin. Infect. Dis. 33, 1387–1 92. doi: 10.1086/322972 11565080

[B2] Kaye K. Pogue J. (2015). Infections Caused by Resistant Gram-Negative Bacteria: Epidemiology and Management. Pharmacotheraphy 35, 949–962. doi: 10.1002/phar.1636 26497481

[B3] Logan L. K. Weinstein R. A. (2017). the Epidemiology of Carbapenem-Resistant *Enterobacteriaceae: The* Impact and Evolution of a Global Menace. J. Infect. Dis. 5, S28–S36. doi: 10.1093/infdis/jiw282 PMC5853342 28375512

[B4] MacNair C. R. Stokes J. M. Carfrae L. A. Fiebig-Comyn A. A. Coombes B. K. Mulvey M. R.. (2018). Overcoming Mcr-1 Mediated Colistin Resistance With Colistin in Combination With Other Antibiotics. Nat. Commun. 9, 458. doi: 10.1038/s41467-018-02875-z 29386620 PMC5792607

[B5] Morris S. Cerceo E. (2020). Trends, Epidemiology, and Management of Multi-Drug Resistant Gram-Negative Bacterial Infections in the Hospitalized Setting. Antibiotics 9, 196. doi: 10.3390/antibiotics904019 PMC7235729 32326058

[B6] Nordmann P. Cuzon G. Naas T. (2009). The Real Threat of *Klebsiella Pneumoniae* Carbapenemase-Producing Bacteria. Lancet Infect. Dis. 9, 228–236. doi: 10.1016/S1473-3099(09)70054- 19324295

[B7] Oliva A. Bianchi A. Russo A. Ceccarelli G. Cancelli F. Aloj F.. (2021). Effect of N-Acetylcysteine Administration on 30-Day Mortality in Critically Ill Patients With Septic Shock Caused by Carbapenem-Resistant Klebsiella Pneumoniae and Acinetobacter Baumannii: A Retrospective Case-Control Study. Antibiotics (Basel) 10 (3), 271. doi: 10.3390/antibiotics10030271 33800296 PMC8001571

[B8] Oliva A. Nguyen B. L. Mascellino M. T. D’Abramo A. Iannetta M. Ciccaglioni A.. (2013). Sonication of Explanted Cardiac Implants Improves Microbial Detection in Cardiac Device Infections. J. Clin. Microbiol. 51 (2), 496–502. doi: 10.1128/JCM.02230-12 23196364 PMC3553873

[B9] Pin L. Xuan L. Mei L. Xuan X. Kewen S. Shuai C.. (2018). Risk Factors for Carbapenem-Resistant *Klebsiella Pneumoniae* Infection: A Meta-Analysis. Microbial Drug Resist. 24, 190–198. doi: 10.1089/mdr.2017.0061 PMC5873294 28749714

[B10] Pitout J. D. Nordmann P. Poirel L. (2015). Carbapenemase-Producing *Klebsiella Pneumoniae*: A Key Pathogen Set for Global Nosocomial Dominance. Antimicrob. Agents Chemother. 59, 5873–5884. doi: 10.1128/AAC.01019-15 26169401 PMC4576115

[B11] Principi N. Silvestri E. Esposito S. (2019). Advantages and Limitations of Bacteriophages for the Treatment of Bacterial Infections. Front. Pharmacol. 10, 513. doi: 10.3389/fphar.2019.00513 31139086 PMC6517696

